# Correction: Novel sensor for the determination of CA 15-3 in serum of breast cancer patients based on Fe–gallic acid complex doped in modified cellulose polymer thin films

**DOI:** 10.1039/d3ra90086j

**Published:** 2023-09-21

**Authors:** Hind A. AlGhamdi, Yasmeen M. AlZahrani, Salha Alharthi, Mohamed S. Mohy-Eldin, Ekram H. Mohamed, Sheta M. Sheta, Said M. El-Sheikh, Safwat A. Mahmoud, Mohamed S. Attia

**Affiliations:** a Chemistry Department, College of Science, Imam Abdulrahman Bin Faisal University P.O. Box 1982 Dammam 31441 Saudi Arabia; b Polymer Materials Research Department, Advanced Technology and New Materials Research Institute (ATNMRI), City of Scientifc Research and Technological Applications (SRTA-City) New Borg El-Arab City, P. O. Box: 21934 Alexandria Egypt; c Analytical Chemistry Department, The British University in Egypt El Sherouk city Cairo 11378 Egypt; d Inorganic Chemistry Department, National Research Centre 33, El-Behouth St., Dokki Giza 12622 Egypt dr.sheta.nrc@gmail.com; e Nanomaterials and Nanotechnology Department, Central Metallurgical R & D Institute Cairo 11421 Egypt selsheikh2001@gmail.com; f Physics Department, Faculty of Science, Northern Border University Arar Saudi Arabia; g Chemistry Department, Faculty of Science, Ain Shams University Abbassia Cairo 11566 Egypt Mohamed_sam@yahoo.com

## Abstract

Correction for ‘Novel sensor for the determination of CA 15-3 in serum of breast cancer patients based on Fe–gallic acid complex doped in modified cellulose polymer thin films’ by Hind A. AlGhamdi *et al.*, *RSC Adv.*, 2023, **13**, 21769–21780, https://doi.org/10.1039/D3RA02495D.

The authors regret the omission of two of the authors, Sheta M. Sheta and Said M. El-Sheikh, from the original manuscript. The corrected author list is as shown above.

The authors regret that the funding information was incorrectly shown in the acknowledgements section of the original manuscript. The corrected funding acknowledgement is as shown below:

The authors extend their appreciation to the Deanship of Scientific Research at Northern Border University, Arar, KSA for funding this research work through the project number NBU-FFR-2023-0051.

The Royal Society of Chemistry apologises for these errors and any consequent inconvenience to authors and readers.

## Supplementary Material

